# Updated systematic review of the effects of exercise on understudied health outcomes in cancer survivors

**DOI:** 10.1002/cam4.6753

**Published:** 2023-11-29

**Authors:** Kathleen M. Sturgeon, Dieuwertje E. Kok, Ian R. Kleckner, Kristin A. Guertin, Jessica McNeil, Traci L. Parry, Diane K. Ehlers, Andrew Hamilton, Kathryn Schmitz, Kristin L. Campbell, Kerri Winters‐Stone

**Affiliations:** ^1^ Department of Public Health Sciences College of Medicine, Penn State University Hershey Pennsylvania USA; ^2^ Division of Human Nutrition and Health Wageningen University & Research Wageningen The Netherlands; ^3^ Department of Pain & Translational Symptom Science, School of Nursing University of Maryland Baltimore Baltimore Maryland USA; ^4^ Department of Public Health Sciences University of Connecticut Health Storrs Connecticut USA; ^5^ Department of Kinesiology, School of Health and Human Sciences University of North Carolina at Greensboro Greensboro North Carolina USA; ^6^ Division of Epidemiology, Department of Quantitative Health Sciences Mayo Clinic Arizona Phoenix Arizona USA; ^7^ Oregon Health & Science University, Library Portland Oregon USA; ^8^ Division of Hematology/Oncology, University of Pittsburgh School of Medicine University of Pittsburgh Pittsburgh Pennsylvania USA; ^9^ Department of Physical Therapy University of British Columbia Vancouver British Columbia Canada; ^10^ Division of Oncological Sciences, School of Medicine Oregon Health & Science University Portland Oregon USA

**Keywords:** bone, cardiovascular, chemotherapy‐induced peripheral neuropathy, cognition, falls, nausea, neoplasm, pain, sexual function, sleep, treatment tolerance

## Abstract

**Introduction:**

The American College of Sports Medicine provided guidelines for exercise prescriptions in cancer survivors for specific cancer‐ and treatment‐related health outcomes. However, there was insufficient evidence to generate exercise prescriptions for 10 health outcomes of cancer treatment. We sought to update the state of evidence.

**Methods:**

We conducted a systematic review of these 10 understudied health outcomes (bone health, sleep, cardiovascular function, chemotherapy‐induced peripheral neuropathy (CIPN), cognitive function, falls and balance, nausea, pain, sexual function, and treatment tolerance) and provided an update of evidence.

**Results:**

While the evidence base for each outcome has increased, there remains insufficient evidence to generate exercise prescriptions. Common limitations observed across outcomes included: variability in type and quality of outcome measurement tools, variability in definitions of the health outcomes, a lack of phase III trials, and a majority of trials investigating breast or prostate cancer survivors only.

**Conclusion:**

We identified progress in the field of exercise oncology for several understudied cancer‐ and treatment‐related health outcomes. However, we were not able to generate exercise prescriptions due to continued insufficient evidence base. More work is needed to prescribe exercise as medicine for these understudied health outcomes, and our review highlights several strategies to aid in research acceleration within these areas of exercise oncology.

## Abstract 2

Marked progress has been made in a short amount of time on understudied cancer‐ and treatment‐related health outcomes. However, compared to the breadth of evidence previously used to generate FITT (frequency, intensity, time, type) prescriptions for anxiety, depressive symptoms, fatigue, health‐related quality of life, lymphedema, and physical function, rigorous research is still warranted. In this paper, we accomplished three goals: (1) presented the state of the evidence for bone health, sleep, cardiovascular function, CIPN, cognitive function, falls and balance, nausea, pain, sexual function, and treatment tolerance; (2) identified common tools used in exercise oncology to assess these specific understudied outcomes, and (3) suggested knowledge gaps that currently limit our ability to provide clinical guidance, or FITT prescriptions, on these understudied outcomes for specialists, patients and caregivers.

## 1 INTRODUCTION

In 2020, there were an estimated 19.3 million new invasive cancer cases diagnosed globally, which translates to over 52,000 cancer diagnoses per day.^1^ The population of cancer survivors is increasing with the survival rates still on the rise and a predicted 28.4 million new cases in 2040.^2^, ^3^ These individuals often face additional physical, mental, or financial challenges due to the cancer or its treatment.

Myriad different cancer treatment regimens are used in cancer patients because of the complexity of cancer.^4^, ^5^ Side effects of cancer treatment regimens often lead to acute and chronic changes in a number of health outcomes, for example through impact on the immune system, gastrointestinal tract, endocrine system, neurological functioning, and many more.^4^ Sequelae related to cancer treatment may eventually lead to, or accelerate, the development of multiple comorbid conditions. One approach to decrease the total burden of cancer‐ and treatment‐related toxicities is through exercise therapy.

The American College of Sports Medicine (ACSM) convened an International Multidisciplinary Roundtable meeting to update guidance on exercise for cancer survivors.^6^ Based on a strong body of evidence, exercise prescriptions were developed for the following cancer‐related health outcomes: anxiety, depressive symptoms, fatigue, health‐related quality of life, and physical function. However, insufficient evidence was available to determine exercise prescriptions for bone health, sleep, cardiovascular function, chemotherapy‐induced peripheral neuropathy (CIPN), cognitive function, falls, nausea, pain, sexual function, and treatment tolerance.^6^ Since the 2019 ACSM publication of the Roundtable recommendations, over 700 randomized controlled trials (RCTs) have been reported in PubMed on exercise in patients with any type of cancer (Medical Subject Headings (MeSH) “exercise” + “neoplasm”). The aim of the present review was to update the state of evidence and, if appropriate, provide evidence‐based FITT (frequency, intensity, time, type) exercise prescriptions. Using search strategies and criteria common to the 2019 ACSM Roundtable Guidelines, we systematically reviewed, evaluated, synthesized, and updated the state of the evidence related to health outcomes for which there was previously insufficient data to issue FITT prescriptions.

## 2 METHODS

A search was conducted in July 2023 using Medline/PubMed, EMBASE, the Cochrane Central Register of Controlled Trials, the Cochrane Collaboration, CINAHL, and the Physical Therapy Evidence Database (PEDro). Standardized search terms were similar to those utilized previously, with differences being related to applicable NCBI updates for MESH terms.^6^ A list of all searches is provided in Supplemental Digital Content 1. The Population, Intervention, Comparison, and Outcome(s) (PICO) criteria were, respectively: cancer survivors (defined as point of diagnosis forward), exercise (nonoccupational/leisure‐time physical activity), standard care/unsupported recommendations, and measurements related to bone health, sleep, cardiovascular function, CIPN, cognitive function, falls, nausea, pain, sexual function, and treatment tolerance. Additional details are provided below.

Screening was conducted by two co‐authors for each outcome, and the group provided resolution for any conflicts. Consistent with the ACSM Roundtable, we focused on traditional modalities of exercise (aerobic, resistance, or combined aerobic + resistance training, with or without balance or flexibility training). Multimodal interventions that incorporated additional components to the exercise component (e.g., diet/nutrition, mindfulness, yoga, physical therapy, pelvic floor exercises) were not included in our review. In addition to the exercise modalities, other eligibility criteria included: published after June 2018 (i.e., date of the first ACSM Roundtable search), a RCT, systematic review or meta‐analysis, adult cancer survivor population (at least 18 years of age), provided results on outcomes related to one of our topic areas, and consisted of exercise training (whereas acute, single bout observations and physical activity behavior change studies were excluded). Only RCTs with at least one experimental arm and one control arm were included. The control group was either defined as “usual care” or “attention control” for the outcomes of interest. Additionally, studies in which doses of exercise components were compared to a control arm consisting of the current ACSM general population recommendations were also included.

For systematic reviews and meta‐analyses published after 2018, individual studies in these types of publications were reviewed against citations in the 2019 Roundtable publication (inclusive of individual studies as well as previous systematic reviews or meta‐analyses). In this way, we documented the total number of participants for each outcome of interest from eligible studies and study cancer site populations (study populations with multiple cancer sites were counted singularly as “mixed”) that have been published to date. Additionally, data collection tools were abstracted. Surveys, scales, or other measurement techniques for each outcome were quantified for frequency of use.

## 3 RESULTS

A total of 78 studies, which included 13,481 cancer survivors, were included in our systematic review across all outcomes. A summary of search returns and systematic review of evidence for each outcome are presented in Table 1. The majority of work was done in breast and prostate cancer patients. Clinical tests, research‐specific objective assessments, and subjective data collection measurement tools were identified for each health outcome and presented in descending order of use (tools listed in order of frequency of utilization) (Table 2). Synthesis of observations across the systematic reviews for individual health outcomes resulted in the identification of recommendations for future clinical trials (Table 3).

**TABLE 1:** Search results and summary of systematic review for 2018–2023 and cumulative evidence up to 2023.

Outcome	2018–2023 returned	2018–2023 title and abstract review	2018–2023 systematic review(s)	2018–2023 RCT(s)	Up to 2023 total sample size	Up to 2023 total cancer sites
Bone health	162	45	2	7	584	2
Sleep	284	58	2	8	3380	9
Cardiovascular function	680	52	1	10	351^a^	2^a^
CIPN	427	219	9	10	683	10
Cognitive function	678	37	4	3	2533	5
Falls and balance	103	47	5	8	1017	8
Nausea	49	12	1	1	937	15
Pain	172	138	6	19	2030	8
Sexual function	198	28	3	3	540	1
Treatment tolerance	265	72	2	9	1426	4

*Note*: Number of search returns (2018–2023), number of articles that passed title and abstract review for the updated search. Numbers after full text review for inclusion as a systematic review or RCT for the updated search. Abstraction represents total number of cancer survivors and cancer study specific sites from prior (up to 2018) and current (2018–2023) systematic review.

^a^ Summary data for vascular function.

**TABLE 2:** Cancer‐related health outcomes and their measurement tools in exercise oncology studies.

Understudied health outcome	Measurement tools identified in the current review
Bone health	Clinical tests: DEXA, CT scan
Sleep	Objective: wrist‐worn accelerometer used for total sleep time and sleep efficiency (ratio of total sleep time to total time in bed X 100)
	Subjective: PSQI
Cardiovascular function	Clinicals tests: LVEF, GLS
	Objective: FMD, PWV, RHI
CIPN	Clinical tests: vibration sense
	Subjective: CIPN‐20, FACT‐NTX, NTSS‐6
Cognitive function	Objective: TMT‐A, TMT‐B
	Subjective: EORTC, FACT‐Cog, PROMIS
Falls and balance	Objective: force plate, physical function tests, Fullerton Advanced Balance Scale, Sensory Organization Test, incidence of falls (self‐reported)
Nausea	Subjective: EORTC QLQ‐C30 symptom domain for nausea/vomiting
Pain	Subjective: Pain numerical rating scale or visual analog scale, BPI, subscales from: EORTC, (LANSS), SPADI shoulder pain, and SF‐36. Pressure pain threshold
Sexual function	Subjective: IIEF for erectile dysfunction, subscales from: EORTC, EPIC
Treatment tolerance	Objective: Chemotherapy completion rates, RDI, treatment modifications

Abbreviations: BPI, brief pain inventory; CIPN, chemotherapy‐induced peripheral neuropathy; CT, computed tomography; DEXA, dual‐energy X‐ray absorptiometry; EORTC, European Organization for Research and Treatment of Cancer; EPIC, Expanded Prostate Cancer Index Composite; FACT‐Cog, functional assessment of cancer therapy—cognition; FACT‐NTX, functional assessment of cancer therapy—neurotoxicity; FMD, flow‐mediated dilation; GLS, global longitudinal strain; IIEF, international index of erectile function; LANSS, Leeds assessment of neuropathic symptoms and signs; LVEF, left ventricular function; PSQI, Pittsburgh sleep quality index; PROMIS, patient‐reported outcomes measurement information system; PWV, pulse wave velocity; RDI, relative dose intensity; RHI, reactive hyperemia index; SF‐36, 36‐item short form survey; SPADI, shoulder pain and disability index; TMT, trail making test A/B.

**TABLE 3:** Exercise oncology study design recommendations for understudied outcomes.

Study component	Action items
Population	Expand study populations to less studied cancer sites
	Identify potential sources of selection bias that could create a ceiling effect (if the study population has limited room for improvement on an outcome)
	Identify potential sources of selection bias for external validity/interpretation (if the study population is not representative of average cancer survivors)
	Define potential interventions or exercises undertaken by “usual care” control groups and/or describe standard care offerings that may differ between hospital sites/healthcare settings/countries
Primary outcome	Leverage appropriate and recommended measurement tools for outcome specificity
	Increase rigor by administering subjective and objective measurements where appropriate
	Implement wearable monitoring for measurement of exercise FITT principles
	Power RCTs to assess understudied outcomes (either as primary or secondary outcomes)
Intervention	Consider multiple study arms for comparison of exercise frequency or intensity
	Conduct comparative effectiveness trials of type of exercise
	Determine how timing of exercise relative to treatment affects outcomes of interest
	A priori define a protocol for documenting exercise adherence with dose modifications or delays

*Note*: Take home messaging suggested to expedite generation of evidence for understudied outcomes.

Abbreviation: FITT, frequency, intensity, time, type.

### 3.1 Bone health

Two new systematic reviews^7^, ^8^, ^9^ and 7 new RCTs^10^, ^11^, ^12^, ^13^, ^14^, ^15^, ^16^ addressed the potential benefits of exercise on bone health (e.g., bone mineral density (BMD) as measured by dual‐energy x‐ray absorptiometry (DEXA) or computed tomography (CT)) in cancer survivors. Measurements were generally collected at baseline and at the end of the study timeline. This evidence is added to the 2 systematic reviews,^17^, ^18^ position paper,^19^ and 3 RCTs identified in the 2019 ACSM Roundtable Guidelines.^6^ Consistent with the prior conclusions in the 2019 ACSM Roundtable Guidelines, moderate‐to‐vigorous intensity resistance training plus high‐impact training (i.e., impact loading exercise that elicits a ground reacting force 3–4 times body weight) performed at least twice per week for at least 12 months remains the most beneficial modality to preserve BMD.^6^, ^15^ Based on new meta‐analyses, benefits may be stronger at the hip than spine and for exercise delivered posttreatment than during treatment.^9^

A handful of studies tested combined programs of moderate‐intensity resistance (i.e., 6–12 RM) and aerobic exercise (i.e., 60%–85% heart rate maximum) performed 2–3 days per week, but these programs neither slowed bone loss nor increased BMD.^10^, ^13^, ^14^, ^15^, ^16^ There is still insufficient evidence to support a benefit of aerobic training alone or resistance training alone (without high‐impact training) on bone outcomes in cancer survivors. Further, as stated in the 2019 ACSM Guidelines, the safety of combined impact + resistance exercise in cancer survivors with osteoporosis or bony metastases has yet to be evaluated and thus, may not be indicated in these subpopulations at this time. It is noteworthy to acknowledge several trials testing a soccer training program that has slowed bone loss in breast and prostate cancer survivors.^20^, ^21^, ^22^ Though a sports‐based intervention may be difficult to prescribe and broadly replicate (and must be weighed against injury risks), the atypical loading from soccer training further suggests that alternative modalities need to be considered for bone health outcomes.

### 3.2 Sleep

One new systematic review^23^ and 8 new RCTs comprising 10 intervention arms were identified.^24^, ^25^, ^26^, ^27^, ^28^, ^29^, ^30^, ^31^ Of the recently published RCTs, 3 focused on aerobic exercise only, 2 conducted a resistance‐only arm, 2 combined aerobic and resistance exercise, 2 used aerobic‐based high‐intensity interval training (HIIT) and 1 used a multimodal HIIT + resistance exercise intervention. Seven studies conducted self‐reported sleep quality measures with the Pittsburgh Sleep Quality Index (PSQI), and one included both subjective and objective sleep measures.^26^ Two of the trials observed significant benefits of exercise (1 mixed HIIT + resistance^25^ and 1 aerobic exercise^29^) on subjective sleep outcomes (PSQI). Conversely, no statistically significant benefits of exercise on sleep were observed in the other trials.^24^, ^26^, ^27^, ^30^, ^31^

Heterogeneity in systematic review assessments were also observed. Fang et al.'s findings are in contrast to Mercier et al., cited in the 2019 ACSM Roundtable Guidelines.^23^, ^32^ While Mercier et al. found no effect of exercise on sleep outcomes among cancer survivors based on 17 RCTs, Fang et al. identified 22 exercise RCTs in cancer patients and reported a small positive effect of exercise to improve total subjective (PSQI‐measured) sleep quality and sleep onset latency, as well as objective (actigraph) measures of sleep onset latency.^23^, ^32^ However, no significant impact on other PSQI measures (daytime dysfunction, sleep disturbance, sleep duration, sleep efficiency, and use of sleep medication) or on objectively measured sleep efficiency was observed. Overall, summary measures of exercise on subjective and objective sleep were mixed. Individual‐level factors such as time of day of exercise and chronotype were not considered in the included studies and systematic reviews and may contribute to the heterogeneity in findings. While evidence is accumulating for beneficial effects of exercise on sleep, there is currently insufficient evidence for a FITT prescription.

### 3.3 Cardiovascular function

A total of 6 studies have examined the effect of exercise training on cardiotoxicity as measured by left ventricular ejection fraction (LVEF) or global strain (GLS) as measured by echocardiography.^33^, ^34^, ^35^, ^36^, ^37^, ^38^ While 2 RCTs provide new evidence since 2019,^35^, ^36^ there remains insufficient evidence to determine the effect of exercise on cardiac function in cancer survivors. However, recent guidance from several clinical entities (e.g., American Heart Association, European Society of Cardiology, and the International Cardio‐Oncology Society) has determined that LVEF is insufficient in evaluation of cardiotoxicity for patients receiving cancer therapy, and a more comprehensive cardiovascular evaluation is required for monitoring of, and management for, cardiovascular toxicities.^39^, ^40^, ^41^ Therefore, given the known impact of exercise on the cardiovascular system, we reviewed the state of the evidence for vascular function in cancer patients.

We identified 4 new RCTs assessing vascular function, as measured by flow‐mediated dilation or vascular pressure waveforms, in cancer patients.^42^, ^43^, ^44^, ^45^ This evidence is added to a meta‐analysis and 2 previous RCTs cited in the 2019 ACSM Guidelines and which examined the effects of exercise on vascular function in cancer survivors using vascular‐specific outcome measures.^46^, ^47^, ^48^ In these 5 RCTs, 3 focused on aerobic exercise,^44^, ^46^, ^47^ 1 on resistance training,^43^ and 2 on combined aerobic and resistance exercise.^42^, ^45^ Approximately half of the patients were prostate cancer survivors and the rest were breast cancer survivors. Overall, there appears to be a moderate effect for improvement in vascular function with aerobic exercise training, in line with noncancer populations. However, most of the studies were small, assessed vascular function as a secondary outcome, and did not report timing of the intervention relative to treatment. Therefore, there remains insufficient evidence to provide a FITT prescription.

### 3.4 CIPN

We identified 9 new systematic reviews^49^, ^50^, ^51^, ^52^, ^53^, ^54^, ^55^, ^56^, ^57^ and 10 new RCTs^58^, ^59^, ^60^, ^61^, ^62^, ^63^, ^64^, ^65^, ^66^, ^67^ in addition to the 3 prior RCTs that were already included in the 2019 ACSM Guidelines.^68^, ^69^, ^70^ Exercise modalities varied considerably between studies (3 used only aerobic, 3 only resistance, 4 aerobic + resistance, and 3 balance + aerobic and/or resistance). A variety of cancer types were studied (breast, gastrointestinal, gynecological, lung, lymphoma, and mixed populations), with the majority of interventions occurring during neurotoxic chemotherapy. Patient‐reported outcomes (e.g., CIPN‐20, FACT‐NTX, single‐symptom numerical rating scales) were the predominant assessment tool.

The systematic reviews concluded that exercise was promising for prevention or treatment of CIPN but not proven due to the low study quality and heterogeneity across studies. In the RCTs, heterogeneity was also observed in that 7 studies found benefits of exercise (effect sizes ranging from 0.2 to 1.7), but 6 studies found no benefit, and 1 study being strictly for feasibility. Overall, we conclude that there is insufficient evidence to judge the effects of exercise on CIPN due to the lack of definitive Phase III trials and the heterogeneity in population, exercise intervention, and outcomes.

### 3.5 Cognitive function

We identified 4 systematic reviews/meta‐analyses focused on exercise training and cognitive function and that reported on new RCTs not included in the 2019 ACSM Guidelines (1 in prostate cancer, 1 focused on HIIT with mixed cancer populations, 1 in breast cancer, 1 including mixed study designs with survivors during chemotherapy).^71^, ^72^, ^73^, ^74^ We also identified 3 new RCTs (1 in chemotherapy‐exposed breast cancer survivors, 1 in rectal cancer survivors during chemoradiation, 1 prehabilitation trial in colorectal cancer survivors).^75^, ^76^, ^77^, ^78^ This evidence is added to the systematic review by Campbell et al.,^6^, ^79^ included in the 2019 ACSM Guidelines. The majority of the evidence is derived from breast cancer patients, supervised settings, and studies measuring cognitive function as a self‐reported, secondary outcome. The vast majority of studies measured cognition via the EORTC QLQ‐C30 (cognition subscale), followed by the FACT‐Cog. Overall, findings suggest the benefits of exercise on self‐reported cognitive function; however, more research is needed to confirm this, as data remain preliminary. Available evidence also indicates that, while HIIT may improve self‐reported cognition, it may not confer additional benefit above and beyond that of moderate‐intensity aerobic exercise.^72^, ^77^

Fewer studies have measured cognition objectively and, among those that have, risk of bias is high, and demonstration of effect is equivocal. Specifically, these studies have provided low‐quality evidence that exercise generally may benefit executive function and attention,^74^ and aerobic exercise may benefit processing speed in breast cancer survivors.^61^, ^63^ To date, only one fully powered trial (a combined aerobic + resistance training intervention in women with breast cancer) has been completed with published results.^75^, ^78^ Researchers observed improvements in self‐reported cognition, but no changes in objectively measured cognition or magnetic resonance imaging‐derived regional brain volume (i.e., hippocampus) compared to the control group. Currently, there is insufficient evidence to develop a FITT prescription for subjectively or objectively measured cognitive function.

### 3.6 Falls

Five systematic reviews^8^, ^80^, ^81^, ^82^, ^83^ and 10 new RCTs^11^, ^63^, ^84^, ^85^, ^86^, ^87^, ^88^, ^89^ were identified. One of these studies had falls as the primary outcome,^89^ and some monitored it as an adverse event via patient report (e.g., Ref. [85]). Estimating the risk of falls remains difficult due to its low incidence. Thus, we expanded this outcome to include studies with balance as an outcome given the potential link between balance impairments and falls.^90^ The one RCTs assessed falls using self‐report (1 study) and balance using: a force plate (2 studies), the Short Physical Performance Battery (2 studies), the Fullerton Advanced Balance Scale (2 studies), the Sensory Organization Test (2 studies), or outcomes related to balance such as timed up and go (4 studies) or 3‐meter walk. Studies were conducted in prostate, breast, abdominal, gynecological, and mixed cancer populations. Exercise modalities varied by intervention such that 3 studies conducted aerobic + resistance training, balance only (1 study), balance + aerobic (2 studies), balance + aerobic + resistance (3 studies), and balance + resistance (3 studies). Collectively, 5 studies found benefits of exercise, 5 studies found no benefits, and no studies found harm of exercise. We conclude there is insufficient evidence to develop a FITT prescription due to heterogeneity in the effect of exercise on falls or balance, and due to variability in exercise interventions and outcomes across studies. However, existing falls prevention approaches may be considered for reducing risk of falls in cancer populations, as suggested previously in the 2019 ACSM Guidelines.^6^, ^80^

### 3.7 Nausea

One new meta‐analysis and 1 RCT were identified. This evidence is added to 1 RCT from the 2019 ACSM Guidelines.^91^, ^92^ Nakano et al. conducted a meta‐analysis focused on the impact of aerobic and resistance exercise on nausea in cancer patients (hematological, breast, and prostate). Four interventions were aerobic only, 4 mixed (aerobic + resistance) interventions, and 2 were resistance‐based exercise. Studies included in the meta‐analyses all used the EORTC QLQ‐C30 to report physical symptoms, including nausea. The meta‐analysis reported no statistically significant difference between the intervention and control groups in nausea symptoms. In addition to the RCTs reviewed by Nakano et al.,^91^ one RCT was identified and found no effect of a moderate‐intensity aerobic exercise intervention on nausea.^92^ In summary, the initial evidence to date suggests that aerobic and/or resistance‐based exercise has limited impact on feelings of nausea and thus does not warrant a FITT prescription at this time. It is worth noting, however, that there is emerging evidence focused on the effects of other intervention types (e.g., yoga, Qigong, Tai chi) on nausea, which would warrant further investigation given that these types of interventions fell outside the scope of our review.

### 3.8 Pain

Six systematic reviews^93^, ^94^, ^95^, ^96^, ^97^ and 19 new RCTs^59^, ^98^, ^99^, ^100^, ^101^, ^102^, ^103^, ^104^, ^105^, ^106^, ^107^, ^108^, ^109^, ^110^, ^111^, ^112^, ^113^, ^114^, ^115^ were newly identified in addition to the 2 prior RCTs in the 2019 ACSM Roundtable Guidelines.^116^, ^117^ Across the 21 RCTs, 12 studies targeted patients with breast cancer, 2 hematologic, 4 were conducted in mixed populations, and one study each focusing on head/neck/thyroid, esophageal, and prostate. The majority of studies were conducted during active treatment (during chemotherapy and/or radiation or biologics, or hormone therapy) or soon after surgery (e.g., for breast, esophageal, or head/neck cancers). Exercise modalities were well‐represented, as 4 studies used only aerobic, 6 studies used only resistance, and 11 studies used aerobic + resistance interventions. Patient‐reported outcomes were the predominant measurement tools, with the QLQ‐C30 Pain Subscale used in 6 studies, pain numerical rating scale in 10 studies, the Brief Pain Inventory in 2 studies, and pain subscales from LEEDS Neuropathic pain, SPADI shoulder pain, or SF‐36 also used in other studies. While 65% of studies suggested a benefit of exercise on pain, there is insufficient evidence to form a FITT prescription. Although 11 studies using aerobic + resistance training spanning 1427 patients were identified, there was heterogeneity in methodological detail as well as observation of an exercise effect/improvement.

### 3.9 Sexual function

A total of 3 new systematic reviews^82^, ^118^, ^119^ and 3 RCTs^120^, ^121^, ^122^ in men with prostate cancer included measures of self‐reported sexual function. Each of the systematic reviews focused on different intervention types. Schumacher et al. focused on aerobic and/or resistance exercise during radiation therapy and reported one study with evidence that exercise leads to improvements in sexual function, but not sexual activity.^118^ Zdravkovic et al. reviewed resistance exercise‐only interventions and reported overall improvements in self‐reported sexual function and sexual activity in the exercise group.^82^ Lastly, Reimer et al. focused on multimodel interventions and reported favorable outcomes in 6 out of 10 exercise‐only RCTs.^119^ Of recently published RCTs, 2 of 3 reported improvements in either sexual function or sexual activity.^120^, ^122^

To date, observations on exercise and sexual function are only in men with prostate cancer. While some studies have looked at the effects of pelvic floor exercises, Pilates and belly dance on sexual function outcomes in women, these were excluded due to the type of exercise intervention (i.e., not aerobic and/or resistance exercise interventions).^123^, ^124^ Therefore, we cannot comment on the effects of aerobic and/or resistance exercise interventions on sexual function outcomes in women. Given some degree of heterogeneity in findings currently available in prostate cancer patients, there is insufficient evidence to generate a FITT prescription. Furthermore, given the multifaceted etiology of sexual dysfunction, it appears promising that exercise could have a beneficial effect. It is also important to recognize that some forms of treatment (e.g., nerve‐sparing surgical procedures, hormonal treatments) can lead to changes in sexual function that cannot be reversed via exercise participation.

### 3.10 Treatment tolerance

Two new systematic reviews^125^, ^126^ and 3 new RCTs^64^, ^127^, ^128^ were identified and added to earlier findings in the 2019 ACSM Roundtable Guidelines. A total of 9 RCTs are now available covering a total of 1426 patients, and all of these RCTs focused on treatment tolerance (as measured by relative dose intensity (RDI), treatment completion rate, or requirement to reduce treatment dose based on original planned treatment) in populations undergoing chemotherapy, some combined with radiotherapy. No studies dedicated to tolerance of other treatments, such as immunotherapy, were identified in the current search. Four of the 9 RCTs focused on breast cancer patients, and 2 studies included colorectal cancer patients, whereas patients with acute myeloid leukemia (AML), lymphoma or mixed populations were studied in the remaining RCTs.

Exercise interventions showed heterogeneity in effect. Although some promising results were described, especially for patients with breast cancer,^129^, ^130^ a substantial number of studies have not been able to confirm a beneficial effect of either aerobic training, exercise training or combined intervention on treatment tolerance. On the other hand, current data do not suggest that exercise would impede treatment tolerance. In line with another systematic literature review published in 2019,^125^ we conclude that there is still insufficient evidence to provide a FITT prescription to improve treatment tolerance.

## 4 DISCUSSION

In this systematic review, we assessed specific health outcomes relevant to cancer survivors that were previously determined to have insufficient evidence in the 2019 ACSM Roundtable Guidelines.^6^ We updated the state of the evidence for the following health outcomes: bone health, sleep, cardiovascular function, CIPN, cognitive function, falls, nausea, pain, sexual function, and treatment tolerance. While the evidence base for these understudied health outcomes has increased, there remains insufficient evidence for a FITT prescription for any outcome. Insufficiency of evidence is based on heterogeneity for an effect of exercise on the outcomes and for the lack of high‐quality RCTs (designed and powered on the outcome of interest at the primary endpoint). Despite promising progress in a relatively short period of time, several limitations and knowledge gaps were identified across outcomes of interest (Table 3).

The majority of evidence is still in the most common cancers, including breast cancer and prostate cancer. However, we did note more diversity of cancer sites in RCTs compared to previous reviews. More studies have included other cancer populations, such as colorectal cancer and hematological malignancies. These trials are important because, while approximately 50% of cancer survivors are breast or prostate cancer survivors, more trials need to address outcomes in the other 50% of cancer survivors of various sites (e.g., lung, colorectal, gynecological, hematologic, bladder, and kidney), so that future FITT prescriptions can be fully generalizable to survivors of any cancer.^131^ Although we found insufficient evidence to develop FITT prescriptions, we anticipate that investigations to determine if differences exist between survivors of different cancer sites in their response to exercise interventions on cancer‐related outcomes will be warranted.

We observed several limitations in the study design that also warrant more research to strengthen the evidence in support of FITT prescriptions. First, few studies examined these understudied cancer‐ and cancer treatment‐related outcomes as a primary outcome. For example, of the RCTs reviewed for cognitive function and falls, the vast majority measured cognition as a secondary outcome. Thus, observations of no differences in cognitive function between groups may be underpowered. Similarly, some studies employed sport‐based interventions that elicited protective effects; however, these studies do not contain clear FITT principles, and therefore, results were difficult to quantify. For example, several trials testing a soccer training program slowed bone loss in breast and prostate cancer survivors.^18^, ^19^, ^20^ While sports‐based interventions may be difficult to prescribe and broadly replicate (and must be weighed against injury risks), the stresses and possible mental and social benefits they provide could be worthwhile.

Secondly, measures employed were not always those recommended for the study of these outcomes. For example, the evidence for self‐reported cognitive function was generally restricted to exercise's effects on the EORTC QLQ‐30 cognition subscale. Outcome‐specific measures (e.g., FACT‐Cog for self‐reported cognition) and measures following consensus recommendations (e.g., International Cognition and Cancer Task Force)^132^ may be more appropriate for the examination of exercise's effects. Similarly, future studies on CIPN should follow recent suggestions for outcome measures,^133^ such as the use of patient‐reported outcomes (specifically the CIPN‐20), clinical assessments (e.g., the Total Neuropathy Score (TNS)), and assessments of physical function (e.g., 6‐minute walk test, handgrip), as well as design considerations for clinical trials studying CIPN.^134^

We also observed that for some cancer‐ and treatment‐related health outcomes, the definition, and therefore measurement, of the outcome is difficult. Specifically, studies assessing falls risk as the primary outcome is difficult due to a low incidence of falls with exercise, but it has been done and new research is underway.^89^, ^135^ However, data on falls as reported for safety monitoring may be used to provide preliminary evidence of the effects of exercise on the incidence of falls. Balance outcomes that predict fall risk might be a more feasible outcome to use to generate a FITT prescription. Thus, outcomes specific to balance (e.g., timed balance tests, force plate metrics) as opposed to functional outcomes that involve balance but are not specific to balance (e.g., timed up and go), are encouraged.

Related to the above measurement issues of outcome specificity is the issue of subjective, patient‐reported outcomes versus objective assessments. The 2019 ACSM Roundtable Guidelines observed strong evidence for a beneficial exercise effect on anxiety, depressive symptoms, fatigue, health‐related quality of life, and self‐reported physical function.^6^ These self‐reported, subjective health outcomes are interrelated. Based on the current state of evidence that we reviewed, we anticipate that there may be a disconnect between observations for an exercise effect on subjective and objective measurements for health outcomes such as sleep, CIPN, and cognitive function. The same outcome, when measured subjectively versus objectively, may not be correlated with itself (e.g., subjective cognitive function seems to be more related to depression and quality of life, while objective cognitive function is more related to demographic and clinical variables).^136^ Therefore, inclusion of both subjective and objective measurement tools for these understudied outcomes is necessary.

Third, among those studies targeting the outcomes of focus in this review as primary, many were pilot/feasibility studies, and/or the study population was often restricted to individuals experiencing the specific outcome of interest. Thus, the magnitude of exercise's benefit may be higher than what can be expected in cancer survivors with minimal or no symptoms. Ultimately, more fully powered trials are needed. These limitations and biases warrant caution in interpretation and synthesis of evidence but also emphasize areas of exercise oncology needing further research. A particular need is to understand these survivorship issues in individuals living with cancer. One funding opportunity includes The National Cancer Institute's (NCI) RFA‐CA‐22‐027, “Research to Understand and Address the Survivorship Needs of Individuals Living with Advanced Cancer.”.

In our systematic review, we identified RCTs that investigated comparisons of exercise training regimens (such as differences in exercise intensity, or combinations of exercise type). Such studies challenge the definition of a control group, such that “usual care” should not be a nonexercise control. These types of intervention arms may become more common as funding agencies request such study designs. Indeed, the recent NCI Exercise and Nutrition Interventions to Improve Cancer Treatment‐Related Outcomes (ENICTO) consortium indicated that nonresponsive applications would propose, “an exercise intervention trial, alone or in combination with medical nutrition, that only compares a general physical activity guideline (PAG) approach to a control condition”. Despite the potential for more complex study designs in the future, we observed that there is still a need for studies to identify the main effects of some types/intensities of training (e.g., resistance training alone, HIIT). In addition to understanding main effects of core exercise modalities, the added benefits of certain types/intensities (e.g., balance training, interval training) when supplementing standard exercise modalities, will also be necessary.

Lastly, we also observed variability in timing of interventions (presurgery vs postsurgery, during chemotherapy or radiation vs after these treatments) across studies, which limits our ability to define FITT prescriptions specific to certain time points along the cancer care continuum. This undoubtedly also contributes substantially to individual patient capacity to adhere to an exercise prescription or to observe benefits of exercise on studied outcomes. Further, during or after treatment, it should also be considered that other unresolved treatment‐related symptoms interfering with function (e.g., the impact of urinary disorders and hot flashes on sleep) may preclude a beneficial impact of exercise interventions (perhaps due to low adherence) on understudied outcomes.^137^

Our work built upon the 2019 ACSM Roundtable's list of cancer‐related health outcomes with high clinical relevance for which exercise may have therapeutic benefit. There may be other cancer‐related health outcomes that are understudied yet can be addressed using exercise (e.g., stress, fear of recurrence). Additionally, while our search criteria were limited to RCTs with exercise training modalities conducive to abstracting an exercise prescription, there are other forms of physical activity that may provide benefit (e.g., sports, yoga, dance). A known limitation of our review was the decision to not include mind–body exercise, sports, or other movement modalities. Indeed, these types of exercise are critical not only for physical and mental health benefits linked with cancer treatment outcomes, but there is also a recognized importance of sustained behavior change and long‐term adherence associated with engagement and participation in these types of exercise.

It is also noted that physical therapy interventions (individualized assessment and programming), were excluded from our review. While physical therapy is a standard of care in many countries, the individualized nature of the care makes it difficult to contextualize the effect of specific doses or modalities on our outcomes of interest. We also made determinations on how quantification of the understudied side effects (and how the understudied outcomes are defined) would be utilized in our synthesis of evidence. Therefore, we have specifically detailed the types of questionnaires, subscales, or objective measures that were observed in our review to be most utilized (Table 2).

## 5 SUMMARY AND CONCLUSIONS

Marked progress has been made in a short amount of time on understudied cancer‐ and treatment‐related health outcomes. However, compared to the breadth of evidence previously used to generate FITT prescriptions for anxiety, depressive symptoms, fatigue, health‐related quality of life, lymphedema, and physical function, rigorous research is still warranted. In this paper, we accomplished three goals: (1) presented the state of the evidence for bone health, sleep, cardiovascular function, CIPN, cognitive function, falls and balance, nausea, pain, sexual function, and treatment tolerance; (2) identified common tools used in exercise oncology to assess these specific understudied outcomes, and (3) suggested knowledge gaps that currently limit our ability to provide clinical guidance, or FITT prescriptions, on these understudied outcomes for specialists, patients and caregivers. This work provides a roadmap for future study design and meta‐analysis considerations that will move the field forward faster for understudied health outcomes in exercise oncology.

## AUTHOR CONTRIBUTIONS

**Kathleen M. Sturgeon:** Conceptualization (lead); data curation (supporting); formal analysis (equal); project administration (equal); writing – original draft (lead); writing – review and editing (equal). **Dieuwertje E. Kok:** Data curation (supporting); formal analysis (supporting); project administration (equal); resources (lead); writing – original draft (supporting); writing – review and editing (supporting). **Ian R. Kleckner:** Data curation (supporting); formal analysis (supporting); writing – original draft (supporting); writing – review and editing (supporting). **Kristin A. Guertin:** Data curation (supporting); formal analysis (supporting); writing – original draft (supporting); writing – review and editing (supporting). **Jessica McNeil:** Data curation (supporting); formal analysis (supporting); writing – original draft (supporting); writing – review and editing (supporting). **Traci L. Parry:** Data curation (supporting); formal analysis (supporting); writing – original draft (supporting); writing – review and editing (supporting). **Diane K. Ehlers:** Data curation (supporting); formal analysis (supporting); writing – original draft (supporting); writing – review and editing (supporting). **Andrew Hamilton:** Data curation (lead); methodology (supporting); resources (equal); writing – review and editing (supporting). **Kathryn Schmitz:** Data curation (supporting); formal analysis (supporting); writing – original draft (supporting); writing – review and editing (supporting). **Kristin L. Campbell:** Formal analysis (supporting); methodology (equal); supervision (equal); writing – original draft (supporting); writing – review and editing (supporting). **Kerri Winters‐Stone:** Data curation (supporting); formal analysis (supporting); methodology (lead); supervision (equal); writing – original draft (supporting); writing – review and editing (equal).

## FUNDING INFORMATION

This work was supported by the National Institutes of Health (K07CA221931 to IRK and R25CA203650). The results of the present study do not constitute endorsement by ACSM. The results of the study are presented clearly, honestly, and without fabrication, falsification, or inappropriate data manipulation.

## CONFLICT OF INTEREST STATEMENT

The authors report no conflicts of interest.

## Supporting information


**Data S1:** Supplemental Digital Content 1.

## Data Availability

Search strategies for replication are provided in Table S1. Additional organizational files used for the systematic review are available upon request to the corresponding author.
