# Supplementary material for: Updated systematic review of the effects of exercise on understudied health outcomes in cancer survivors
Source: Cancer Med. 2023 Nov 29;12(24):22278–92. doi: 10.1002/cam4.6753 (PMC10757127; doi:10.1002/cam4.6753)
Supplement: Supplementary file 1 — Data S1: Supplemental Digital Content 1. [file CAM4-12-22278-s001.docx]

Supplemental Methods - Search Strategies

Database: Ovid MEDLINE(R) ALL <1946 to July 13, 2023>

**Bone Health**

1. exp Neoplasms/

2. exp cancer survivors/

3. exp antineoplastic protocols/

4. exp antineoplastic agents/

5. exp radiotherapy/

6. 1 or 2 or 3 or 4 or 5

7. exp EXERCISE/

8. exp Physical Fitness/

9. exp Exercise Therapy/

10. exp EXERCISE MOVEMENT TECHNIQUES/

11. 7 or 8 or 9 or 10

12. 6 and 11

13. exp Muscle Strength/

14. exp Physical Endurance/

15. 13 or 14

16. 6 and 15

17. exp Physical Exertion/

18. 6 and 17

19. ((cancer* or neoplas* or carcinom* or adenocarcino* or tumor* or tumour* or malig* or metasta* or leukem* or leukaem* or lymphom* or melanom*) adj7 ((physical* adj3 (fit* or strength* or stamin* or endur* or capac*)) or (exercis* adj3 (aerobic* or therap* or treat* or capac* or tolera* or capab*)))).mp. [mp=title, abstract, original title, name of substance word, subject heading word, floating sub-heading word, keyword heading word, protocol supplementary concept word, rare disease supplementary concept word, unique identifier, synonyms]

20. 12 or 16 or 18 or 19

21. exp Body Constitution/

22. exp "Body Weights and Measures"/

23. exp Overnutrition/

24. exp OSTEOPOROSIS/

25. exp Bone Density/

26. exp FRACTURES, BONE/

27. 21 or 22 or 23 or 24 or 25 or 26

28. 20 and 27

29. limit 28 to (english language and humans)

30. limit 29 to (meta analysis or systematic reviews)

31. limit 29 to (controlled clinical trial or comparative study or randomized controlled trial)

32. 31 not 30

33. 29 not (31 or 32)

**Sleep**

1 exp Neoplasms/ (3703867)

2 exp cancer survivors/ (7436)

3 exp antineoplastic protocols/ (155811)

4 exp antineoplastic agents/ (1205966)

5 exp radiotherapy/ (202346)

6 1 or 2 or 3 or 4 or 5 (4375015)

7 exp EXERCISE/ (233111)

8 exp Physical Fitness/ (35026)

9 exp Exercise Therapy/ (60035)

10 exp EXERCISE MOVEMENT TECHNIQUES/ (9766)

11 7 or 8 or 9 or 10 (296341)

12 6 and 11 (11349)

13 exp Muscle Strength/ (41911)

14 exp Physical Endurance/ (36498)

15 13 or 14 (75320)

16 6 and 15 (1913)

17 12 or 16 (12468)

18 exp Physical Exertion/ (57302)

19 6 and 18 (743)

20 17 or 19 (13085)

21 20 not 17 (617)

22 limit 21 to humans (484)

23 ((cancer* or neoplas* or carcinom* or adenocarcino* or tumor* or tumour* or malig* or metasta* or leukem* or leukaem* or lymphom* or melanom*) adj7 ((physical* adj3 (fit* or strength* or stamin* or endur* or capac*)) or (exercis* adj3 (aerobic* or therap* or treat* or capac* or tolera* or capab*)))).mp. [mp=title, abstract, original title, name of substance word, subject heading word, floating sub-heading word, keyword heading word, organism supplementary concept word, protocol supplementary concept word, rare disease supplementary concept word, unique identifier, synonyms] (1633)

24 20 or 23 (13477)

25 exp SLEEP/ (92291)

26 exp Sleep Wake Disorders/ (103381)

27 exp Chronobiology Phenomena/ (106458)

28 exp MELATONIN/ (22053)

29 exp SNORING/ (4515)

30 exp Polysomnography/ (24123)

31 25 or 26 or 27 or 28 or 29 or 30 (271700)

32 24 and 31 (280)

33 (sleep* or rapid eye mov* or somnol* or circadian* or chronobio* or melatonin or insomn* or parasomn* or dyssomn* or snore* or snoring or restless leg* or (periodic* adj3 leg* adj3 mov*)).mp. [mp=title, abstract, original title, name of substance word, subject heading word, floating sub-heading word, keyword heading word, organism supplementary concept word, protocol supplementary concept word, rare disease supplementary concept word, unique identifier, synonyms] (353118)

34 24 and 33 (530)

35 32 or 34 (535)

36 (201806* or 201807* or 201808* or 201809* or 20181* or 2019* or 202*).ed. (4792085)

37 35 and 36 (234)

38 limit 37 to (english language and humans) (229)

**Cardiovascular function**

1 exp Neoplasms/ (3694385)

2 exp cancer survivors/ (7320)

3 exp antineoplastic protocols/ (155499)

4 exp antineoplastic agents/ (1203712)

5 exp radiotherapy/ (202008)

6 1 or 2 or 3 or 4 or 5 (4364326)

7 exp EXERCISE/ (232278)

8 exp Physical Fitness/ (34935)

9 exp Exercise Therapy/ (59810)

10 exp EXERCISE MOVEMENT TECHNIQUES/ (9731)

11 7 or 8 or 9 or 10 (295363)

12 6 and 11 (11278)

13 exp Muscle Strength/ (41709)

14 exp Physical Endurance/ (36439)

15 13 or 14 (75063)

16 6 and 15 (1903)

17 12 or 16 (12390)

18 exp Physical Exertion/ (57290)

19 6 and 18 (743)

20 17 or 19 (13007)

21 20 not 17 (617)

22 limit 21 to humans (484)

23 ((cancer* or neoplas* or carcinom* or adenocarcino* or tumor* or tumour* or malig* or metasta* or leukem* or leukaem* or lymphom* or melanom*) adj7 ((physical* adj3 (fit* or strength* or stamin* or endur* or capac*)) or (exercis* adj3 (aerobic* or therap* or treat* or capac* or tolera* or capab*)))).mp. [mp=title, abstract, original title, name of substance word, subject heading word, floating sub-heading word, keyword heading word, organism supplementary concept word, protocol supplementary concept word, rare disease supplementary concept word, unique identifier, synonyms] (1615)

24 20 or 23 (13390)

25 exp Cardiovascular Diseases/ (2622441)

26 exp Cardiovascular System/ (1322833)

27 exp Cardiovascular Physiological Phenomena/ (1009557)

28 exp ENDOTHELIAL CELLS/ (72752)

29 25 or 26 or 27 or 28 (3654237)

30 (((heart* or cardi* or myocardi* or endothel*) adj7 (function* or dysfunction* or weak* or strong* or strength* or capac* or diseas* or fail* or capab* or incapab* or enhanc* or interfer* or prevent* or promot* or support*)) or metabolic syndrom*).mp. [mp=title, abstract, original title, name of substance word, subject heading word, floating sub-heading word, keyword heading word, organism supplementary concept word, protocol supplementary concept word, rare disease supplementary concept word, unique identifier, synonyms] (1186183)

31 29 or 30 (3945162)

32 24 and 31 (2188)

33 (201806* or 201807* or 201808* or 201809* or 20181* or 2019* or 202*).ed. (4740477)

34 32 and 33 (619)

35 limit 34 to (english language and humans) (557)

36 limit 35 to (meta analysis or systematic reviews) (59)

37 limit 35 to (controlled clinical trial or comparative study or randomized controlled trial) (109)

38 35 not (36 or 37) (390)

**CIPN**

1 exp Neoplasms/ (3694385)

2 exp cancer survivors/ (7320)

3 exp antineoplastic protocols/ (155499)

4 exp antineoplastic agents/ (1203712)

5 exp radiotherapy/ (202008)

6 1 or 2 or 3 or 4 or 5 (4364326)

7 exp EXERCISE/ (232278)

8 exp Physical Fitness/ (34935)

9 exp Exercise Therapy/ (59810)

10 exp EXERCISE MOVEMENT TECHNIQUES/ (9731)

11 7 or 8 or 9 or 10 (295363)

12 6 and 11 (11278)

13 exp Muscle Strength/ (41709)

14 exp Physical Endurance/ (36439)

15 13 or 14 (75063)

16 6 and 15 (1903)

17 12 or 16 (12390)

18 exp Physical Exertion/ (57290)

19 6 and 18 (743)

20 17 or 19 (13007)

21 20 not 17 (617)

22 limit 21 to humans (484)

23 ((cancer* or neoplas* or carcinom* or adenocarcino* or tumor* or tumour* or malig* or metasta* or leukem* or leukaem* or lymphom* or melanom*) adj7 ((physical* adj3 (fit* or strength* or stamin* or endur* or capac*)) or (exercis* adj3 (aerobic* or therap* or treat* or capac* or tolera* or capab*)))).mp. [mp=title, abstract, original title, name of substance word, subject heading word, floating sub-heading word, keyword heading word, organism supplementary concept word, protocol supplementary concept word, rare disease supplementary concept word, unique identifier, synonyms] (1615)

24 20 or 23 (13390)

25 exp Peripheral Nervous System Diseases/ (160771)

26 exp Peripheral Nervous System/ (463986)

27 exp Nervous System Physiological Phenomena/ (1137313)

28 25 or 26 or 27 (1561758)

29 24 and 28 (1017)

30 (neuropath* or neurotox* or ((neur* or nerv*) adj3 (pathol* or disease* or damag* or dysfunction* or injur* or traum* or toxic*))).mp. [mp=title, abstract, original title, name of substance word, subject heading word, floating sub-heading word, keyword heading word, organism supplementary concept word, protocol supplementary concept word, rare disease supplementary concept word, unique identifier, synonyms] (608728)

31 24 and 30 (342)

32 29 or 31 (1174)

33 (201806* or 201807* or 201808* or 201809* or 20181* or 2019* or 202*).ed. (4740477)

34 32 and 33 (350)

**Cognitive function**

1 exp Neoplasms/ (3694385)

2 exp cancer survivors/ (7320)

3 exp antineoplastic protocols/ (155499)

4 exp antineoplastic agents/ (1203712)

5 exp radiotherapy/ (202008)

6 1 or 2 or 3 or 4 or 5 (4364326)

7 exp EXERCISE/ (232278)

8 exp Physical Fitness/ (34935)

9 exp Exercise Therapy/ (59810)

10 exp EXERCISE MOVEMENT TECHNIQUES/ (9731)

11 7 or 8 or 9 or 10 (295363)

12 6 and 11 (11278)

13 exp Muscle Strength/ (41709)

14 exp Physical Endurance/ (36439)

15 13 or 14 (75063)

16 6 and 15 (1903)

17 12 or 16 (12390)

18 exp Physical Exertion/ (57290)

19 6 and 18 (743)

20 17 or 19 (13007)

21 20 not 17 (617)

22 limit 21 to humans (484)

23 ((cancer* or neoplas* or carcinom* or adenocarcino* or tumor* or tumour* or malig* or metasta* or leukem* or leukaem* or lymphom* or melanom*) adj7 ((physical* adj3 (fit* or strength* or stamin* or endur* or capac*)) or (exercis* adj3 (aerobic* or therap* or treat* or capac* or tolera* or capab*)))).mp. [mp=title, abstract, original title, name of substance word, subject heading word, floating sub-heading word, keyword heading word, organism supplementary concept word, protocol supplementary concept word, rare disease supplementary concept word, unique identifier, synonyms] (1615)

24 20 or 23 (13390)

25 exp Cognition Disorders/ (109755)

26 exp memory disorders/ (32084)

27 exp Mental Processes/ (1162079)

28 exp neuropsychological tests/ (189635)

29 ((think* or thought* or cognit* or memor* or mind* or brain* or cereb* or mental*) adj5 (function* or capac* or abilit* or capab* or process* or dysfunction* or inabilit* or unable or incapab* or impair* or reduc* or interfer* or test or tests or testing or tested)).mp. [mp=title, abstract, original title, name of substance word, subject heading word, floating sub-heading word, keyword heading word, organism supplementary concept word, protocol supplementary concept word, rare disease supplementary concept word, unique identifier, synonyms] (573267)

30 25 or 26 or 27 or 28 or 29 (1672285)

31 24 and 30 (1032)

32 (chemobrain* or chemo-brain*).mp. (346)

33 31 or 32 (1367)

34 (201806* or 201807* or 201808* or 201809* or 20181* or 2019* or 202*).ed. (4740477)

35 33 and 34 (551)

**Falls and balance**

1 exp Neoplasms/ (3694385)

2 exp cancer survivors/ (7320)

3 exp antineoplastic protocols/ (155499)

4 exp antineoplastic agents/ (1203712)

5 exp radiotherapy/ (202008)

6 1 or 2 or 3 or 4 or 5 (4364326)

7 exp EXERCISE/ (232278)

8 exp Physical Fitness/ (34935)

9 exp Exercise Therapy/ (59810)

10 exp EXERCISE MOVEMENT TECHNIQUES/ (9731)

11 7 or 8 or 9 or 10 (295363)

12 6 and 11 (11278)

13 exp Muscle Strength/ (41709)

14 exp Physical Endurance/ (36439)

15 13 or 14 (75063)

16 6 and 15 (1903)

17 12 or 16 (12390)

18 exp Physical Exertion/ (57290)

19 6 and 18 (743)

20 17 or 19 (13007)

21 20 not 17 (617)

22 limit 21 to humans (484)

23 ((cancer* or neoplas* or carcinom* or adenocarcino* or tumor* or tumour* or malig* or metasta* or leukem* or leukaem* or lymphom* or melanom*) adj7 ((physical* adj3 (fit* or strength* or stamin* or endur* or capac*)) or (exercis* adj3 (aerobic* or therap* or treat* or capac* or tolera* or capab*)))).mp. [mp=title, abstract, original title, name of substance word, subject heading word, floating sub-heading word, keyword heading word, organism supplementary concept word, protocol supplementary concept word, rare disease supplementary concept word, unique identifier, synonyms] (1615)

24 20 or 23 (13390)

25 exp Accidental Falls/ (27428)

26 exp POSTURAL BALANCE/ (26916)

27 exp MUSCLE WEAKNESS/ (9316)

28 ((accident* adj5 (fall* or fell)) or ((lose* or losing or lost or unsteady or instab* or weak* or frail* or unable or inabilit* or cannot or fail*) adj5 (stand* or walk* or equilibri* or balanc*))).mp. (52366)

29 25 or 26 or 27 or 28 (83634)

30 24 and 29 (256)

31 (201806* or 201807* or 201808* or 201809* or 20181* or 2019* or 202*).ed. (4740477)

32 30 and 31 (93)

33 limit 32 to (english language and humans) (89)

34 limit 33 to (meta analysis or systematic reviews) (6)

35 limit 33 to (comparative study or controlled clinical trial or randomized controlled trial) (16)

36 35 not 34 (16)

37 33 not (34 or 35) (67)

**Nausea**

1 exp Neoplasms/ (3694385)

2 exp cancer survivors/ (7320)

3 exp antineoplastic protocols/ (155499)

4 exp antineoplastic agents/ (1203712)

5 exp radiotherapy/ (202008)

6 1 or 2 or 3 or 4 or 5 (4364326)

7 exp EXERCISE/ (232278)

8 exp Physical Fitness/ (34935)

9 exp Exercise Therapy/ (59810)

10 exp EXERCISE MOVEMENT TECHNIQUES/ (9731)

11 7 or 8 or 9 or 10 (295363)

12 6 and 11 (11278)

13 exp Muscle Strength/ (41709)

14 exp Physical Endurance/ (36439)

15 13 or 14 (75063)

16 6 and 15 (1903)

17 12 or 16 (12390)

18 exp Physical Exertion/ (57290)

19 6 and 18 (743)

20 17 or 19 (13007)

21 20 not 17 (617)

22 limit 21 to humans (484)

23 ((cancer* or neoplas* or carcinom* or adenocarcino* or tumor* or tumour* or malig* or metasta* or leukem* or leukaem* or lymphom* or melanom*) adj7 ((physical* adj3 (fit* or strength* or stamin* or endur* or capac*)) or (exercis* adj3 (aerobic* or therap* or treat* or capac* or tolera* or capab*)))).mp. [mp=title, abstract, original title, name of substance word, subject heading word, floating sub-heading word, keyword heading word, organism supplementary concept word, protocol supplementary concept word, rare disease supplementary concept word, unique identifier, synonyms] (1615)

24 20 or 23 (13390)

25 exp Nausea/ (21320)

26 exp Vomiting/ (33140)

27 25 or 26 (39862)

28 24 and 27 (34)

29 (nausea* or vomit* or emes* or emet* or (throw* adj up)).mp. [mp=title, abstract, original title, name of substance word, subject heading word, floating sub-heading word, keyword heading word, organism supplementary concept word, protocol supplementary concept word, rare disease supplementary concept word, unique identifier, synonyms] (123951)

30 24 and 29 (100)

31 28 or 30 (101)

32 (201806* or 201807* or 201808* or 201809* or 20181* or 2019* or 202*).ed. (4740477)

33 31 and 32 (42)

**Pain**

1 exp Neoplasms/ (3694385)

2 exp cancer survivors/ (7320)

3 exp antineoplastic protocols/ (155499)

4 exp antineoplastic agents/ (1203712)

5 exp radiotherapy/ (202008)

6 1 or 2 or 3 or 4 or 5 (4364326)

7 exp EXERCISE/ (232278)

8 exp Physical Fitness/ (34935)

9 exp Exercise Therapy/ (59810)

10 exp EXERCISE MOVEMENT TECHNIQUES/ (9731)

11 7 or 8 or 9 or 10 (295363)

12 6 and 11 (11278)

13 exp Muscle Strength/ (41709)

14 exp Physical Endurance/ (36439)

15 13 or 14 (75063)

16 6 and 15 (1903)

17 12 or 16 (12390)

18 exp Physical Exertion/ (57290)

19 6 and 18 (743)

20 17 or 19 (13007)

21 20 not 17 (617)

22 limit 21 to humans (484)

23 ((cancer* or neoplas* or carcinom* or adenocarcino* or tumor* or tumour* or malig* or metasta* or leukem* or leukaem* or lymphom* or melanom*) adj7 ((physical* adj3 (fit* or strength* or stamin* or endur* or capac*)) or (exercis* adj3 (aerobic* or therap* or treat* or capac* or tolera* or capab*)))).mp. [mp=title, abstract, original title, name of substance word, subject heading word, floating sub-heading word, keyword heading word, organism supplementary concept word, protocol supplementary concept word, rare disease supplementary concept word, unique identifier, synonyms] (1615)

24 20 or 23 (13390)

25 exp PAIN/ (436451)

26 exp Pain Management/ (39333)

27 exp Pain Measurement/ (93161)

28 exp ANALGESIA/ (47623)

29 exp Analgesics/ad, ae, tu, to [Administration & Dosage, Adverse Effects, Therapeutic Use, Toxicity] (278361)

30 25 or 26 or 27 or 28 or 29 (711977)

31 24 and 30 (679)

32 (201806* or 201807* or 201808* or 201809* or 20181* or 2019* or 202*).ed. (4740477)

33 31 and 32 (188)

34 limit 33 to (english language and humans) (172)

35 limit 34 to (meta analysis or systematic reviews) (19)

36 limit 34 to (comparative study or controlled clinical trial or randomized controlled trial) (54)

37 36 not 35 (54)

38 34 not (35 or 36) (99)

**Sexual function**

1 exp Neoplasms/ (3694385)

2 exp cancer survivors/ (7320)

3 exp antineoplastic protocols/ (155499)

4 exp antineoplastic agents/ (1203712)

5 exp radiotherapy/ (202008)

6 1 or 2 or 3 or 4 or 5 (4364326)

7 exp EXERCISE/ (232278)

8 exp Physical Fitness/ (34935)

9 exp Exercise Therapy/ (59810)

10 exp EXERCISE MOVEMENT TECHNIQUES/ (9731)

11 7 or 8 or 9 or 10 (295363)

12 6 and 11 (11278)

13 exp Muscle Strength/ (41709)

14 exp Physical Endurance/ (36439)

15 13 or 14 (75063)

16 6 and 15 (1903)

17 exp Physical Exertion/ (57290)

18 6 and 17 (743)

19 ((cancer* or neoplas* or carcinom* or adenocarcino* or tumor* or tumour* or malig* or metasta* or leukem* or leukaem* or lymphom* or melanom*) adj7 ((physical* adj3 (fit* or strength* or stamin* or endur* or capac*)) or (exercis* adj3 (aerobic* or therap* or treat* or capac* or tolera* or capab*)))).mp. [mp=title, abstract, original title, name of substance word, subject heading word, floating sub-heading word, keyword heading word, organism supplementary concept word, protocol supplementary concept word, rare disease supplementary concept word, unique identifier, synonyms] (1615)

20 12 or 16 or 18 or 19 (13390)

21 exp Sexual Dysfunction, Physiological/ (32428)

22 exp Sexual Behavior/ (118011)

23 exp Reproductive Physiological Phenomena/ (1500581)

24 exp Genitalia/ (581477)

25 exp INFERTILITY/ (71367)

26 exp reproductive techniques/ (162983)

27 21 or 22 or 23 or 24 or 25 or 26 (2036416)

28 20 and 27 (821)

29 (((sex* or reproduc*) adj3 (activ* or behav* or desir* or interest* or health* or function* or dysfunction* or capac* or incapac* or capab* or incapab* or desir* or fertil*)) or infertil* or impoten* or vaginismus or dyspareun*).mp. (332639)

30 20 and 29 (247)

31 28 or 30 (948)

32 (201806* or 201807* or 201808* or 201809* or 20181* or 2019* or 202*).ed. (4740477)

33 31 and 32 (190)

34 limit 33 to (english language and humans) (164)

35 limit 34 to (meta analysis or systematic reviews) (21)

36 limit 34 to (controlled clinical trial or comparative study or randomized controlled trial) (33)

37 34 not (35 or 36) (110)

**Treatment tolerance**

1 exp Neoplasms/ (3694385)

2 exp cancer survivors/ (7320)

3 exp antineoplastic protocols/ (155499)

4 exp antineoplastic agents/ (1203712)

5 exp radiotherapy/ (202008)

6 1 or 2 or 3 or 4 or 5 (4364326)

7 exp EXERCISE/ (232278)

8 exp Physical Fitness/ (34935)

9 exp Exercise Therapy/ (59810)

10 exp EXERCISE MOVEMENT TECHNIQUES/ (9731)

11 7 or 8 or 9 or 10 (295363)

12 6 and 11 (11278)

13 exp Muscle Strength/ (41709)

14 exp Physical Endurance/ (36439)

15 13 or 14 (75063)

16 6 and 15 (1903)

17 exp Physical Exertion/ (57290)

18 6 and 17 (743)

19 ((cancer* or neoplas* or carcinom* or adenocarcino* or tumor* or tumour* or malig* or metasta* or leukem* or leukaem* or lymphom* or melanom*) adj7 ((physical* adj3 (fit* or strength* or stamin* or endur* or capac*)) or (exercis* adj3 (aerobic* or therap* or treat* or capac* or tolera* or capab*)))).mp. [mp=title, abstract, original title, name of substance word, subject heading word, floating sub-heading word, keyword heading word, organism supplementary concept word, protocol supplementary concept word, rare disease supplementary concept word, unique identifier, synonyms] (1615)

20 12 or 16 or 18 or 19 (13390)

21 limit 20 to (english and human) (11332)

22 exp Blood Cell Count/ (149888)

23 exp respiratory physiological phenomena/ or exp respiration/ (243174)

24 exp Respiration Disorders/ (209837)

25 exp Anemia/ (171211)

26 exp Oxygen Consumption/ (109094)

27 exp Drug Administration Schedule/ (105619)

28 exp neutropenia/ (19898)

29 (Blood cell* or blood count* or breathless or anemi* or anaemi* or dyspne* or ((short* or troubl* or difficult* or problem*) adj2 breath*)).mp. (450272)

30 (neutropeni* or ((chemother* or chemoradiat*) adj5 (complet* or success* or finish* or fail* or suspend* or terminat*) adj7 (rate or rates or statistic* or percent*))).mp. [mp=title, abstract, original title, name of substance word, subject heading word, floating sub-heading word, keyword heading word, organism supplementary concept word, protocol supplementary concept word, rare disease supplementary concept word, unique identifier, synonyms] (50298)

31 22 or 23 or 24 or 25 or 26 or 27 or 28 or 29 or 30 (1198107)

32 21 and 31 (901)

33 (201806* or 201807* or 201808* or 201809* or 20181* or 2019* or 202*).ed. (4740477)

34 32 and 33 (232)

35 limit 34 to (meta analysis or systematic reviews) (24)

36 limit 32 to (controlled clinical trial or randomized controlled trial) (200)

37 36 not 35 (200)

38 32 not (35 or 36) (677)
